# S. Driggs

**Published:** 1862-01

**Authors:** S. Driggs

**Affiliations:** Lexington, Ky.


LEXINGTON, KY., Jan. 21, 1862.

DR. TAFT—*Dear Sir:*—According to promise I herewith send you a set of the castings which I have designed and used, to obtain, in an easy and practical manner, a zinc die, by pouring into the plaster impression.

The advantages of obtaining a zinc cast by pouring into the impression are too patent to require a favorable word. Every dentist has ofttimes felt the insufficiency of sand, however good, or moulding flask, however ingenious, to meet a large class of cases which must be fitted equally well with others of more favorable form. The effort to accomplish this has resulted in the construction of these rings, by means of which I am able to procure in each and every case a metallic die, with uniform accuracy—the most unfavorable with the same facility and exactness as the most favorable.

The method of procedure is as follows: Take impression with plaster of Paris in the usual way, without feldspar or sand, using the ordinary impression cup. Remove the impression from the cup by inverting and rapping gently with a small mallet or hammer, letting it fall upon a towel to prevent mutilation. Place ring No. 1 upon a table, and pour into it a batter of plaster and sand, equal parts, until about half full; press the impression down into this until the highest point of it shall be level with the top of the ring. After it has set trim off any superfluous portion, and place in a stove oven. When thoroughly dry remove from the oven and place upon it ring No. 2, (of this there are two sizes; use the one nearest the size of the impression,) and immediately pour into it your zinc, pouring at its minimum liquefied temperature. When it has become sufficiently cool to handle remove ring No. 1 and the impression, and turning ring No. 2, containing the die, face upward; place upon it ring No. 3 and it is ready to receive the molten lead.

Allow me, in conclusion, to submit the following as some of the advantages to be derived by the use of the rings:

No particular form of impression cup is essential.

No feldspar or sand is necessary, or even desirable, in the impression; plaster of Paris alone producing a better impression, besides being more agreeable to both patient and operator. The plaster and sand into which it is imbeded in the ring effectually prevents the accidents, or changes in form which might otherwise occur.

The rings are cast, and are of such size and shape and thickness that the most desirable size and form is given to both die and counter die.

Much actual time and labor is saved. The liability of soiling fingers or clothes is avoided, and, though the case be such as would cause great trouble and much time, if moulded in sand, the temper of your mechanical assistant is not jeopardized, or his patience exhausted.

I have not found the forge a suitable place for drying the impression, but the heat of a cooking stove oven, which is even and never too intense, is well adapted to the purpose.

Respectfully, etc., S. Driggs.

